# Rapid purification of gold nanorods for biomedical applications

**DOI:** 10.1016/j.mex.2014.07.007

**Published:** 2014-08-09

**Authors:** Federica Scaletti, Chang Soo Kim, Luigi Messori, Vincent M. Rotello

**Affiliations:** a Department of Chemistry “Ugo Schiff”, University of Florence, Via della Lastruccia 3-13, 50019 Sesto Fiorentino, Florence, Italy; b Department of Chemistry, University of Massachusetts, 710 North Pleasant Street, Amherst, MA 01003, USA

**Keywords:** Selective separation of gold nanorods from by-products, Gold nanorods, Centrifugation, Nanoparticle, Shape separation, Purification, Surface plasmon resonance

## Abstract

Small gold nanorods (GNRs) with longitudinal plasmon absorption in the near-infrared window (700–900 nm) are of great interest for *in vivo* optical applications (*e.g.*, photothermal therapy) and for their high-payload-to-carrier ratio for drug delivery. Common synthetic strategies for GNR production afford spherical and cubical nanoparticles in addition to the desired GNRs. Thus, several methods have been proposed for the selective separation of GNRs from the reaction by-products. For example, centrifugation has been used to separate the high aspect ratio (AR) GNRs (AR > 4). However, it is difficult to separate small sized GNRs with low AR (AR ≤ 4) that are particularly promising for biomedical applications. Here, we describe a simple and fast procedure for the separation of small GNRs with AR of 4, and length of 28 nm from reaction by-products.

The shape separation is achieved through centrifugation according to the following steps:

• Isolation of all gold products of the reaction from the excess of cetyltrimethylammonium bromide through a first cycle of centrifugation.
• Optimization of the speed and the time of centrifugation for the separation of GNRs from the reaction by-products.
• Shape separation of GNRs through a second cycle of centrifugation.

The effectiveness of this procedure is documented.

## Method

The final reaction mixture for GNRs contains the desired GNRs, a variety of gold nanoparticles (NPs) of different shapes (spherical, cubic, *etc.*). To obtain an homogeneous population of GNRs, two cycles of centrifugation were carried out. After the synthesis, all reaction products (spheres, cubes, and rods) were first isolated and purified from the excess of cetyltrimethylammonium bromide (CTAB) through centrifugation at high rpm. CTAB needs to be removed as much as possible for many applications owing to its cytotoxicity [1]. Then, shape separation was afforded through a second cycle of centrifugation adapted from the method reported by Sharma et al. [2] and optimized for the size of interest.

Due to the large shape-dependent differences in the respective sedimentation velocities, in the course of centrifugation, spheres and cubes were sedimented at the bottom of the centrifuge tube while most GNRs remained in the supernatant solution. Therefore, an efficient shape separation can be achieved by tuning the centrifugation times and speeds.

## Method details

### Step 1: GNR synthesis

#### Materials

Sodium borohydride (>98%), cetyltrimethylammonium bromide (>99%), and silver nitrate (99.5%) were purchased from Acros Organics. Chloroauric acid (99.9%) was purchased from Strem Chemical, and L-(+)-ascorbic acid was purchased from Alfa Aesar. All chemicals were used as received. All the solutions were prepared with deionized water produced by a Millipore System.

#### Procedure

GNRs, with AR of 3.9 ± 0.5 (width = 7.5 ± 0.8 nm, length = 28.6 ± 3.5 nm) were synthesized by a seed-mediated method according to Nikoobakht et al. [3] and Ratto et al. [4] with slight modifications. After the synthesis, the structural and optical properties of GNRs were characterized by Transmission Electron Microscopy (TEM) and spectrophotometric analysis. TEM images were acquired on a JEOL 200FX with a tungsten filament operating at 200 kV. Optical extinction spectra were recorded using a Molecular Devices SpectraMax M2 plate reader.

### Step 2: GNR separation from CTAB

The GNRs were separated from excess CTAB and then purified from the by-products through centrifugation with an Eppendorf centrifuge 5810R using 2 ml centrifuge tubes. During centrifugation, temperature was kept at 27 °C to avoid the crystallization of CTAB that may affect the separation.

#### Procedure

- Synthesized GNRs were placed in centrifuge tubes (1.5 ml of GNRs for each centrifuge tube) and centrifuged at the rate of 10,630 rpm (12,000 × g) for 30 min. After centrifugation, precipitates can be clearly seen at the bottom of the centrifuge tubes.
- The supernatant was carefully removed with a pipet, leaving a volume of ∼50 μl solution with the precipitates.
- The precipitates were resuspended by addition of 1.45 ml of Milli-Q water, leading to a final volume of 1.5 ml.
- The resuspended GNRs were then combined (Sample A).

### Step 3: Shape separation

- The isolated products from the first centrifugation were placed in centrifuge tubes (1.5 ml for each centrifuge tube) and centrifuged at the rate of 6000 rpm (3824 *g*) for 15 min.
- The supernatant (GNRs) was carefully collected with a pipet (Sample B), leaving ∼50 μl of the solution with the precipitates in the centrifuge tubes.
- The precipitates at the bottom (spheres and cubes) in the 50 μl of the solution were resuspended by the addition of 1.45 ml of Milli-Q water, leading to a final volume of 1.5 ml.
- All the resuspended GNRs were then combined (Sample C).

After separation, the colors of the solutions of the three samples were clearly different as shown in Fig. 1. Moreover, the structural and optical properties of the separated NPs (Sample B and C) were characterized by optical extinction spectra and TEM images, compared to Sample A (Figs. 2 and 3).

The absorption spectrum relative to Sample A (Fig. 2) showed a transversal surface plasmon peak with a maximum at 514 nm and a broadening at longer wavelength, indicating the presence of GNRs and spherical and cubical NPs as well. The absorption spectrum of Sample C showed that, after the separation, the maximum of the surface plasmon peak was at 522 nm while the spectrum relative to Sample B showed a transversal surface plasmon peak with a maximum at 510 nm. These differences indicated the loss of spherical and cubical NPs in Sample B while the bulk of the by-products were in Sample C. Moreover, the longitudinal surface plasmon peak with a maximum at 800 nm in the absorption spectra of Sample C indicated that some GNRs were present as well. The size and shape of the reaction products (Sample A) and separated NPs (Sample B and Sample C) are shown in the TEM images (Fig. 3). As a result, Sample B (Fig. 3B) was comprised mainly of GNRs while Sample C (Fig. 3C) contained mostly spherical NPs, confirming the shape separation by the two steps of centrifugation.

### Step 4: Quantification of the separation

The quantification of the separation was estimated as follow:

- The sizes of 300 NPs (spheres, cubes and GNRs) for each sample were measured with ImageJ software: spherical NPs showed the average diameter of 28.6 ± 4.7 nm, cubes showed the average side length of 14.1 ± 2.9 nm while GNRs showed a width of 7.5 ± 0.8 nm and length of 28.6 ± 3.5 nm.
- The volumes of spherical, cubical NPs and GNRs were calculated; to calculate the volume of GNRs, we assumed that GNRs have a shape of a cylinder capped with two half-spheres.
- The quantification of the separation was expressed in terms of volume percentage of by-products (spheres and cubes) or GNRs respect to the total volume of all the reaction products (Fig. 4).

Before separation, Sample A was comprised of 41.5% of by-products and 58.5% of GNRs (Fig. 4A). After separation, Sample B contained 79.4% of GNRs and 20.6% of by-products while Sample C consisted 14.2% of GNRs and 85.8% of by-products. As shown in Fig. 1, it is important to notice that the sizes (and the volumes) of most by-products were larger than the size of GNRs, implying that even a small amount of by-products greatly affect the total volume of the sample.

## Additional information

### Background

Among the various gold NPs, gold nanorods (GNRs) have attracted particular attention due to their unique properties (structural, optical, and electronic) [5,6]. Moreover, facile and well-established synthetic routes (*e.g*., seed-mediated growth protocol [7]) permit to produce stable structures of GNRs with high yield and monodispersity.

Rod-shaped NPs exhibit two distinct surface plasmon resonances (SPRs): the transversal mode in the visible region and the longitudinal one in the upper visible or near-infrared part of the spectrum [8]. The longitudinal SPR can be tailored to a particular wavelength [9]. There is a linear relationship between the absorption maximum of the longitudinal SPR band and the mean AR of GNRs, which can be tuned during the synthesis [3]. Due to the SPR, GNRs strongly absorb light, converting absorbed light into localized heat. The strong absorption combined with the facile structural tuning property of GNRs is useful in potential *in vivo* applications. (*e.g*., photothermal treatment [10] in the near-infrared window (700–900 nm) where tissue absorption is minimal, along with deeper penetration depth [11]).

GNRs can be functionalized with a variety of small molecules (*e.g*., drugs, imaging probes, and targeting moieties) leading to a wide range of real or potential biomedical applications, both in diagnosis [12] (*e.g*., optical microscopy [13], photo-acoustic imaging [14], biosensing [15]) and therapy (*e.g*., drug delivery [16] and cancer therapeutics [13]).

The common synthetic method of GNRs, such as the seed-mediated method [7], produces a variety of gold spheres and cubes as by-products. Several methods such as selective precipitation [17,18] or oxidation [19], size exclusion chromatography [20], electrophoresis [21], and centrifugation [2,22], have been reported for the purification of high AR (AR > 4), relatively big sized GNRs (width ≥10 nm). However, it is difficult to separate small sized GNRs while there is an increasing demand of small GNRs because of their strong absorption properties [23] and their high-payload-to-carrier ratio (*e.g*., drug delivery) [16]. Here, we optimized the method to allow the separation of small GNRs from reaction by-products with two cycles of centrifugation. This method does not require the addition of surfactant or salts. Moreover, a large amount of GNRs can be easily obtained from by-products with a high yield.

## Figures and Tables

**Fig. 1:**
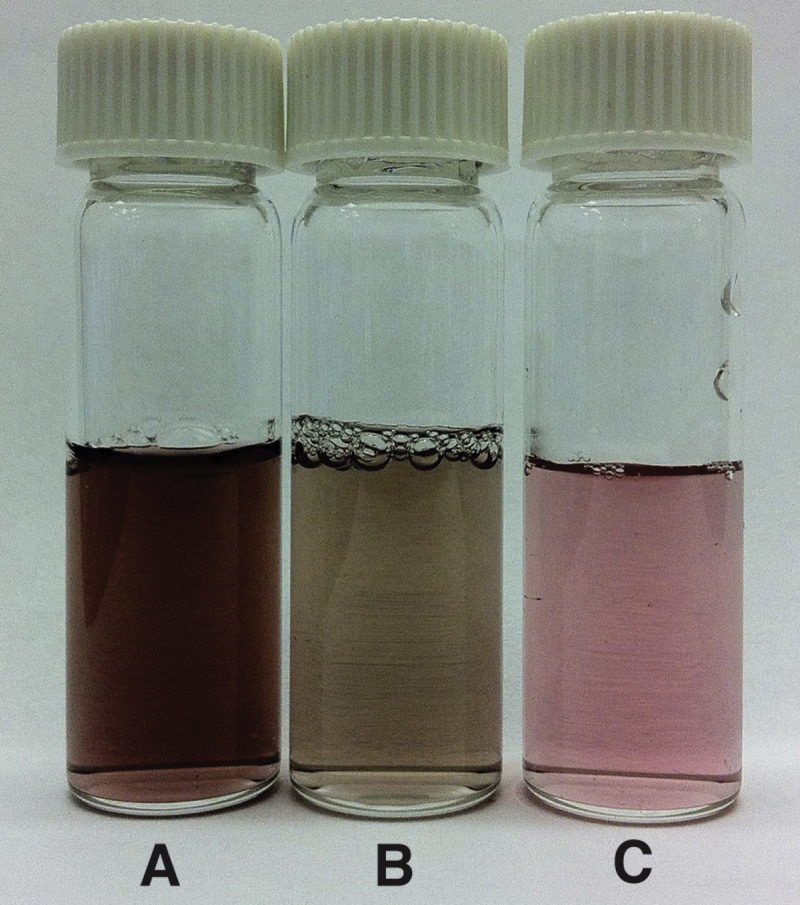
Separation of gold nanorods from reaction products. The figure shows the reaction products after the first centrifugation (A), the supernatant after the shape separation (B), and the resuspended precipitate (C).

**Fig. 2:**
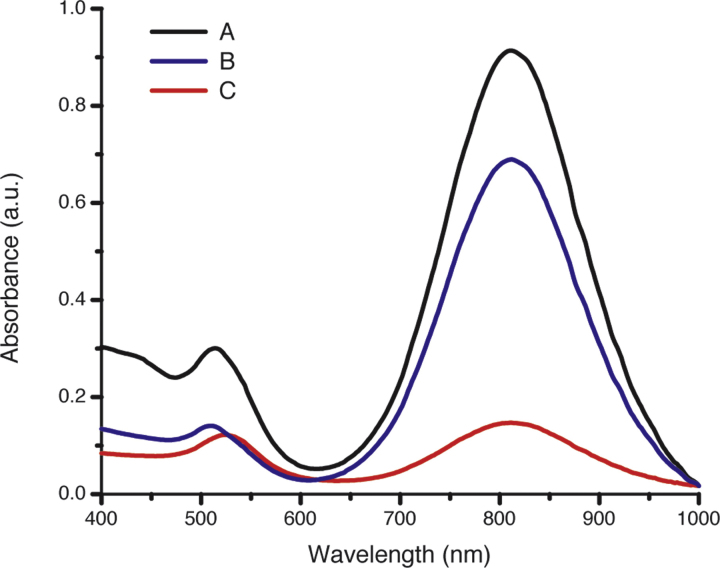
Absorption spectra of reaction products after the first centrifugation (A), the supernatant after the shape separation (B), and the resuspended precipitate (C).

**Fig. 3:**
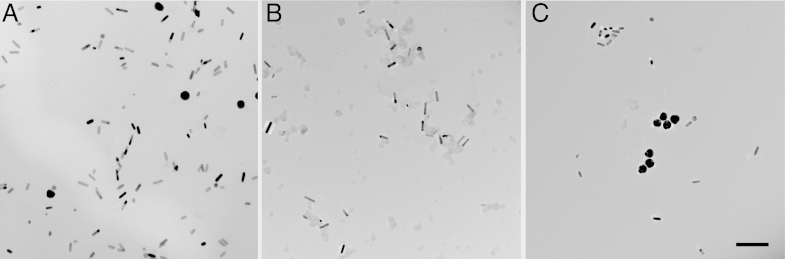
TEM images of reaction products after the first centrifugation (A), the supernatant after the shape separation (B), and the resuspended precipitate (C). Scale bar: 100 nm.

**Fig. 4:**
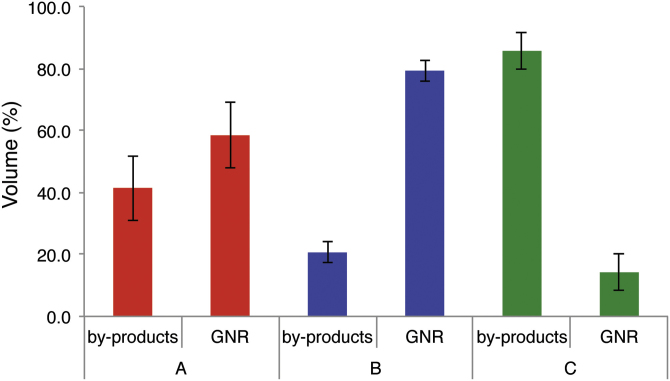
Volume percentage of by-products (spheres and cubes) and GNRs in the sample after the first centrifugation (A), in the supernatant after the shape separation (B), and in the resuspended precipitates (C).

## References

[1] Wang S., Lu W., Tovmachenko O., Ray U.S., Yu H., Ray P.C. (2008). Challenge in understanding size and shape dependent toxicity of gold nanomaterials in human skin keratinocytes. Chem. Phys. Lett..

[2] Sharma V., Park K., Srinivasarao M. (2009). Shape separation of gold nanorods using centrifugation. Proc. Natl. Acad. Sci. U.S.A..

[3] Nikoobakht B., El-Sayed M.A. (2003). Preparation and growth mechanism of gold nanorods (NRs) using seed-mediated growth method. Chem. Mater..

[4] Ratto F., Matteini P., Rossi F., Pini R. (2010). Size and shape control in the overgrowth of gold nanorods. J. Nanopart. Res..

[5] Eustis S., El-Sayed M.A. (2006). Why gold nanoparticles are more precious than pretty gold: noble metal surface plasmon resonance and its enhancement of the radiative and nonradiative properties of nanocrystals of different shapes. Chem. Soc. Rev..

[6] Perez-Juste J., Pastoriza-Santos I., Liz-Marzan L.M., Mulvaney P. (2005). Gold nanorods: synthesis, characterization and applications. Coord. Chem. Rev..

[7] Jana N.R., Gearheart L., Murphy C.J. (2001). Seed-mediated growth approach for shape-controlled synthesis of spheroidal and rod-like gold nanoparticles using a surfactant template. Adv. Mater..

[8] Garcia M.A. (2011). Surface plasmons in metallic nanoparticles: fundamentals and applications. J. Phys. D: Appl. Phys..

[9] Murphy C.J., Orendorff C.J. (2005). Alignment of gold nanorods in polymer surfaces. Adv. Mater..

[10] Dickerson E.B., Dreaden E.C., Huang X., El-Sayed I.H., Chu H., Pushpanketh S., McDonald J.F., El-Sayed M.A. (2008). Gold nanorod assisted near-infrared plasmonic photothermal therapy (PPTT) of squamous cell carcinoma in mice. Cancer Lett..

[11] Weissleder R. (2001). A clear vision for in vivo imaging. Nat. Biotechnol..

[12] Murphy C.J., Gole A.M., Hunyadi S.E., Stone J.W., Sisco P.N., Alkilany A., Kinard B.E., Hankins P. (2008). Chemical sensing and imaging with metallic nanorods. Chem. Commun..

[13] Huang X., El-Sayed I.H., Quian W., El-Sayed M.A. (2006). Cancer cell imaging and photothermal therapy in the near-infrared region by using gold nanorods. J. Am. Chem. Soc..

[14] Mallidi S., Larson T., Aaron J., Sokolov K., Emelianov S. (2007). Molecular specific optoacoustic imaging with plasmonic nanoparticles. Opt. Express.

[15] Aslan K., Lakowicz J.R., Geddes C.D. (2005). Plasmon light scattering in biology and medicine: new sensing approaches, visions and perspectives. Curr. Opin. Chem. Biol..

[16] Wei Q., Ji J., Shen J. (2008). Synthesis of near-infrared responsive gold nanorod/PNIPAAm core/shell nanohybrids via surface initiated ATRP for smart drug delivery. Macromol. Rapid. Commun..

[17] Jana N.R. (2003). Nanorod shape separation using surfactant assisted self-assembly. Chem. Commun..

[18] Park K., Koerner H., Vaia R.A. (2010). Depletion-induced shape and size selection of gold nanoparticles. Nano Lett..

[19] Khanal B.P., Zubarev E.R. (2008). Purification of high aspect ratio gold nanorods: complete removal of platelets. J. Am. Chem. Soc..

[20] Wei G.T., Liu F.K., Chris Wang C.R. (1999). Shape separation of nanometer gold particles by size-exclusion chromatography. Anal. Chem..

[21] Hanauer M., Pierrat S., Zins I., Lotz A., Sonnichsen C. (2007). Separation of nanoparticles by gel electrophoresis according to size and shape. Nano Lett..

[22] Akbulut O., Mace C.R., Martinez R.V., Kumar A.A., Nie Z., Patton M.R., Whitesides G.M. (2012). Separation of nanoparticles in aqueous multiphase systems through centrifugation. Nano Lett..

[23] Mackey M.A., Ali M.R.K., Austin L.A., Near R.D., El-Sayed M.A. (2014). The most effective gold nanorod size for plasmonic photothermal therapy: theory and in vitro experiments. J. Phys. Chem. B.

